# Antioxidant Activity of Fluoxetine and Vortioxetine in a Non-Transgenic Animal Model of Alzheimer’s Disease

**DOI:** 10.3389/fphar.2021.809541

**Published:** 2021-12-24

**Authors:** Giuseppe Caruso, Margherita Grasso, Annamaria Fidilio, Sebastiano Alfio Torrisi, Nicolò Musso, Federica Geraci, Maria Rosaria Tropea, Anna Privitera, Fabio Tascedda, Daniela Puzzo, Salvatore Salomone, Filippo Drago, Gian Marco Leggio, Filippo Caraci

**Affiliations:** ^1^ Department of Drug and Health Sciences, University of Catania, Catania, Italy; ^2^ Oasi Research Institute—IRCCS, Troina, Italy; ^3^ Department of Biomedical and Biotechnological Sciences, University of Catania, Catania, Italy; ^4^ Center for Neuroscience and Neurotechnology, University of Modena and Reggio Emilia, Modena, Italy; ^5^ Department of Life Sciences, University of Modena and Reggio Emilia, Modena, Italy

**Keywords:** oxidative stress, Alzheimer’s disease, depression, amyloid-β, vortioxetine, fluoxetine, TGF-β1, neuroprotection

## Abstract

Depression is a risk factor for the development of Alzheimer’s disease (AD). A neurobiological and clinical continuum exists between AD and depression, with neuroinflammation and oxidative stress being involved in both diseases. Second-generation antidepressants, in particular selective serotonin reuptake inhibitors (SSRIs), are currently investigated as neuroprotective drugs in AD. By employing a non-transgenic AD model, obtained by intracerebroventricular (i.c.v.) injection of amyloid-β (Aβ) oligomers in 2-month-old C57BL/6 mice, we recently demonstrated that the SSRI fluoxetine (FLX) and the multimodal antidepressant vortioxetine (VTX) reversed the depressive-like phenotype and memory deficits induced by Aβ oligomers rescuing the levels of transforming growth factor-β1 (TGF-β1). Aim of our study was to test FLX and VTX for their ability to prevent oxidative stress in the hippocampus of Aβ-injected mice, a brain area strongly affected in both depression and AD. The long-term intraperitoneal (i.p.) administration of FLX (10 mg/kg) or VTX (5 and 10 mg/kg) for 24 days, starting 7 days before Aβ injection, was able to prevent the over-expression of inducible nitric oxide synthase (iNOS) and NADPH oxidase 2 (Nox2) induced by Aβ oligomers. Antidepressant pre-treatment was also able to rescue the mRNA expression of glutathione peroxidase 1 (Gpx1) antioxidant enzyme. FLX and VTX also prevented Aβ-induced neurodegeneration in mixed neuronal cultures treated with Aβ oligomers. Our data represent the first evidence that the long-term treatment with the antidepressants FLX or VTX can prevent the oxidative stress phenomena related to the cognitive deficits and depressive-like phenotype observed in a non-transgenic animal model of AD.

## Introduction

Alzheimer’s disease (AD) represents a type of dementia affecting memory, global cognitive function, and behavior, severe enough to interfere with activities of daily living (Kumar et al., 2021). This disease also presents neuropsychiatric symptoms, such as depression, along with neurodegeneration, neuroinflammation, and oxidative stress phenomena (Caruso et al., 2021). The latter occurs when the homeostatic equilibrium between pro-oxidants species and antioxidants is missing, with the pro-oxidants being in excess (Caruso et al., 2019a). With regard to depression, a neurobiological and clinical continuum has been demonstrated between this disease and AD (Caraci et al., 2018b). In fact, depression represents a risk factor for AD development, while the occurrence of depressive symptoms significantly increases the conversion from mild cognitive impairment (MCI) into AD (Petersen et al., 2014).

It is now well-known that amyloid-β (Aβ), the peptide involved in the pathogenesis of AD, can undergo aggregation, starting with soluble monomers and forming species characterized by higher molecular weight such as oligomers, protofibrils, and mature fibrils (Brorsson et al., 2010). Among the above species, oligomers represent the most toxic species of Aβ, leading to synaptic loss and neuronal death in AD brain (Klein, 2013). It has been shown that oxidative stress plays a crucial role in mediating the toxicity of Aβ oligomers; in fact, neurodegeneration and neuroinflammation as well as the impairment of synaptic plasticity are, at least in part, due to the oxidative stress Aβ oligomers-induced (Varadarajan et al., 2000; Gelain et al., 2012). Oxidative stress is able to promote Aβ oligomerization (Zhao and Zhao, 2013) and β- and γ-secretase activation, the two enzymes involved in the formation of the different Aβ species (Zhao and Zhao, 2013). Markers of oxidative stress have been found in AD animal models before plaques deposition as well as in brain, plasma, and erythrocytes from MCI and AD patients (Glenner and Wong, 1984; Minati et al., 2009), suggesting that redox imbalance and oxidative damage play a key role in an early stage of AD pathophysiology, as well as in the disease progression (Cheignon et al., 2018).

Different groups are currently studying antidepressants in AD (Lozupone et al., 2018). Second-generation antidepressants have been associated with a reduced risk of developing AD, but there are still no clear findings demonstrating the ability of these drugs to counteract the progression of this disease (Correia and Vale, 2021). Positive outcomes have been observed by using selective serotonin reuptake inhibitors (SSRIs) (Dafsari and Jessen, 2020). Long-term SSRI treatment (>4 years) was significantly associated with a delayed progression from MCI to AD (Bartels et al., 2018). The immune regulatory effect of antidepressants observed in depressed patients is attributable to their ability to decrease the levels of pro-inflammatory cytokines (e.g., tumor necrosis factor-α (TNF-α)) and increase those of anti-inflammatory cytokines, such as transforming growth factor-β1 (TGF-β1) (Szałach et al., 2019). Antidepressant drugs could also exert their therapeutic effect by suppressing the production of reactive oxygen and nitrogen species, ROS and RNS respectively, and/or rescuing the antioxidant defense (Behr et al., 2012; Wu et al., 2013). The SSRI fluoxetine (FLX) is able to revert the brain oxidative damage by reducing lipid peroxidation at hippocampal level, also increasing the activity of antioxidant enzymes, such as superoxide dismutase (SOD) and catalase (CAT), in different animal models of depression (Chung et al., 2010; Moretti et al., 2012). Evidence also exists that FLX prevents amyloid pathology, also reverting memory impairment in different animal models of AD (Wang et al., 2014; Jin et al., 2017). Furthermore, FLX exerts neuroprotection in an established *in vitro* model of Aβ-induced neurodegeneration *via* a paracrine signaling mediated by TGF-β1 (Caraci et al., 2016). Acute and long-term treatments with the new multimodal antidepressant vortioxetine (VTX) improve cognitive function in preclinical models of depression (Bennabi et al., 2019). This drug also exhibits an increased efficacy, compared to FLX, in aged mice in counteracting depressive-like behavior and memory deficits (Li et al., 2017; Bennabi et al., 2019). At clinical level, VTX has proven more effective than SSRIs in the treatment of specific clinical domains, such as cognitive deficits in elderly depressed patients (McIntyre et al., 2016; Thase et al., 2016), underlining its therapeutic potential for the treatment of cognitive impairment in depression (Bennabi et al., 2019). Interestingly, VTX exerts antioxidant activity and anti-inflammatory effects in human monocytes/macrophages stimulated with phorbol 12-myristate 13-acetate (PMA), also inducing the shift of macrophages from M1 (pro-inflammatory) to M2 (anti-inflammatory) phenotype (Talmon et al., 2018).

Intracerebroventricular (i.c.v.) injection of Aβ oligomers in mice has been used to obtain a non-transgenic (non-Tg) AD model characterized by memory deficits and depressive-like phenotype (Ledo et al., 2016; Ledo et al., 2020). An equivalent outcome has been observed in rats that underwent i.c.v. injection of Aβ oligomers (Colaianna et al., 2010; Schiavone et al., 2017). By using this non-Tg AD model, we have recently demonstrated that FLX or VTX revert the behavioral and memory alterations induced by Aβ oligomers (Torrisi et al., 2019). In the same study we also detected a significant reduction of the synaptic proteins synaptophysin and PSD-95 paralleled by a significant deficit of TGF-β1 at hippocampal level that was completely rescued by the long-term treatment with FLX or VTX.

Starting from these grounds, we hypothesized that the i.c.v. injection of Aβ oligomers could also induce oxidative stress in the hippocampus of our non-Tg model of AD and that a long-term treatment with FLX or VTX could prevent this phenomenon by regulating the subtle equilibrium between pro- and antioxidant factors.

## Materials and Methods

### Materials

All chemicals and reagents used in this study were of analytical grade and obtained from Sigma-Aldrich Inc. (St. Louis, MO, United States) or Thermo Fisher Scientific Inc. (Pittsburgh, PA, United States) unless specified otherwise.

### Establishment of the Non-Tg AD Mouse Model

The cohorts of animals whose tissues were used for gene and protein analysis are the same described in Torrisi et al. (2019).

Eight-week-old male C57BL/6 mice, obtained from Envigo RMS s.r.l. laboratories (San Pietro al Natisone, Italy), were maintained and used as previously described (Torrisi et al., 2019), following procedures in accordance with the U.K. Animals (Scientific Procedures) Act, 1986 and associated guidelines, EU Directive 2010/63/EU for animal experiments.

As previously described, in order to obtain the non-Tg AD mouse model, 2 µL of the 10 μM Aβ oligomers solution (Bachem Distribution Services GmbH, Weil am Rhein, Germany), prepared according to the original protocol of Klein’s group (Gong et al., 2003), were i.c.v. injected by using a microsyringe with a 28-gauge stainless-steel needle 3.0-mm-long (Hamilton). The injection of this Aβ solution corresponds to 20 pmol of Aβ monomer equivalent, giving a final concentration of approximately 0.18 μg/g tissue.

### Drug Treatment

Vortioxetine hydrobromide [purity > 98.0% (HPLC)] was obtained from H. Lundbeck A/S (Denmark) according to the MTA N.417394 signed by University of Catania (Department of Drug and Health Sciences) and H. Lundbeck A/S and Lundbeck Italia S.p.A. FLX and VTX were prepared and administered i.p. (FLX at 10 mg/kg; VTX at 5 or 10 mg/kg) daily for a total of 21 to 26 days starting from 7 days before Aβ i.c.v injection as previously described in details (Torrisi et al., 2019). Control animals received the vehicle i.p. A total of five groups of animals were employed in this study and are indicated as follows: 1) control group (phosphate-buffered saline [PBS + vehicle (VEH)]; 2) Aβ group (Aβ + VEH); 3) FLX10 group (Aβ + FLX 10 mg/kg); 4) VTX5 group (Aβ + VTX 5 mg/kg); 5) VTX10 group (Aβ + VTX 10 mg/kg). PBS and VEH were injected i.c.v. and i.p., respectively, while to test the drug activity *per se*, so in absence of Aβ, drugs were administered i.p. for a total of 21 days.

### Gene Expression Analysis by Quantitative Real-Time PCR (qRT-PCR)

Gene expression analysis by qRT-PCR was carried out on hippocampal samples. The protocol employed for these experiments is the same previously described (Caruso et al., 2019c; Fidilio et al., 2021) with slight modifications. Briefly, NanoDrop® ND-1000 (Thermo Fisher Scientific, Waltham, MA, United States) was used to determine the RNA concentrations, while Qubit® 3.0 Fluorometer (Thermo Fisher Scientific) was employed to assess the quality of RNA (Fresta et al., 2020a). The reverse transcription was obtained by using the SuperScript III First-Strand Synthesis SuperMix kit. The quantification of all the cDNA samples obtained and loaded in a 384-well plate was measured through a LightCycler® 480 System (Roche Molecular Systems, Inc., Pleasanton, CA, United States). The information relative to the Quanti Tect Primer Assays (Qiagen, Hilden, Germany) used is reported in Table 1. Sample amplification, fluorescence data collection, and sample quantification is the same previously described elsewhere (Caruso et al., 2019c; Fidilio et al., 2021). The relative RNA expression level for each sample was calculated using the 2^−ΔΔCT^ method in which the threshold cycle (CT) value of the target gene is compared to the CT value of the selected internal control (GAPDH gene in our case). The number of samples analyzed obtained by each animal group is indicated in the pertinent Figure legend.

**TABLE 1:** List of primers used for quantitative real-time PCR (qRT-PCR).

Official name^a^	Official symbol	Alternative titles/symbols	Detected transcript	Amplicon length	Cat. No.^b^
Nitric oxide synthase 2, inducible	Nos2	iNOS; Nos-2; Nos2a; i-NOS; NOS-II; MAC-NOS	NM_010927	118 bp	QT00100275
Cytochrome b-245, beta polypeptide	Cybb	Cgd; Cyd; Nox2; C88302; gp91-1; gp91phox; CGD91-phox	NM_007807	146 bp	QT00139797
			XM_006527565		
Glutathione peroxidase 1	Gpx1	Gpx; CGPx; GPx-1; GSHPx-1; AI195024; AL033363	NM_008160	133 bp	QT01195936
Glyceraldehyde-3-phosphate dehydrogenase	Gapdh	Gapd	NM_008084	144 bp	QT01658692
			XM_001003314		
			XM_990238		
			NM_001289726		

a https://www.ncbi.nlm.nih.gov/gene/.

b https://www.qiagen.com/it/shop/pcr/real-time-pcr-enzymes-and-kits/two-step-qrt-pcr/quantitect-primer-assays/.

### Protein Expression Analysis by Western Blot (WB)

WB analysis was performed on hippocampal samples following the previously described procedure (Caraci et al., 2015). Briefly, once the protein concentration in tissue homogenate was determined (Pierce™ BCA protein assay kit), 30 µg of total proteins were denatured, separated by gel electrophoresis, and transferred to nitrocellulose membranes. The membranes were incubated overnight (4°C) with the following primary antibodies: rabbit anti-iNOS (Abcam ab136918, 1:1,000), rabbit anti-Nox2/gp91phox (Abcam ab80508, 1:4,000), rabbit anti-Gpx1 (Cell Signaling Technology 3206, 1:500), mouse anti-β-actin (Sigma Aldrich A4700, 1:1,000). Secondary goat anti-rabbit labeled with IRDye 800 (Li-COR Biosciences; 1:15,000) and goat anti-mouse labeled with IRDye 680 (Li-COR Biosciences; 1:15,000) were used at room temperature in the dark for 1 h after three washes in tris-buffered saline (TBS)/Tween 20× 0.1%. Hybridization signals were detected by the Odyssey Infrared Imaging System (LI-COR Biosciences) and the densitometry analysis was performed by using ImageJ software. The number of samples analyzed obtained by each animal group is indicated in the pertinent Figure legend.

### Mixed Neuronal Cultures

Mixed neuronal cultures consisting of 35–40% neurons and 60–65% glial cells (astrocytes and microglia) were obtained from rats at embryonic day 15 (Harlan Laboratories, Italy) as previously described (Caraci et al., 2016). Cells were grown into DMEM/F12 (1:1) (American Type Culture Collection (ATCC), Manassas, VA, United States) supplemented with 10% horse serum, 10% fetal calf serum, 2 mM glutamine, and 6 mg/ml glucose. After 7–10 days *in vitro*, to avoid the proliferation of non-neuronal elements, cytosine-D-arabinoside (10 µM) was added, for a total of 3 days. Cells were then moved into a maintenance medium in absence of serum. Mixed neuronal cultures were treated with Aβ oligomers (2 µM) for 48 h both in absence or presence of FLX (1 µM) or increasing concentrations of VTX (100 nM, 250 nM, or 1 µM) (pre-treatment of 1 h). The Aβ oligomers-induced toxicity was quantitatively assessed by trypan blue exclusion assay. Cell counts were performed in three to four random microscopic fields/well.

### Statistics

Data are reported as mean ± standard error of the mean (S.E.M.) except in the case of cell experiments in which standard deviation (S.D.) was showed. One-way analysis of variance (ANOVA) followed by Tukey’s *post hoc* test were used for multiple comparisons. The version 8.0 of GraphPad Prism software® (GraphPad, La Jolla, CA, United States) was used to perform all the analyses. Only two-tailed *p* values < 0.05 were considered statistically significant.

### Study Approval

The study was authorized by the Institutional Animal Care and Use Committee (IACUC) of the University of Catania and by the Italian Ministry of Health (DDL 26/2014 and previous legislation; OPBA Project #266/2016). Animal care followed Italian (D.M. 116192) and EEC (O.J. of E.C.L 358/1 12/18/1986) regulations on protection of animals used for experimental and scientific purposes.

## Results

### Fluoxetine and Vortioxetine Decreased the Expression of iNOS and Nox2 mRNAs

**FIGURE 1:**
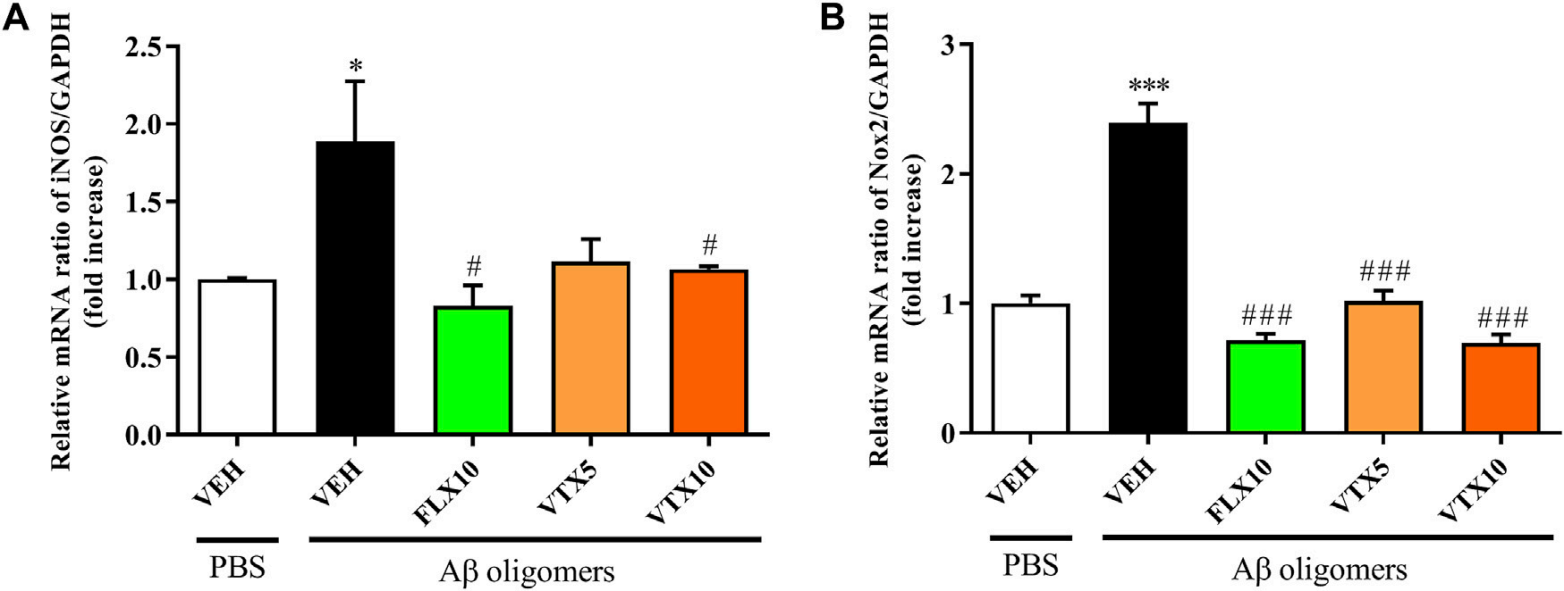
Fluoxetine and vortioxetine decrease the expression of iNOS and Nox2 mRNAs. Effects induced by i.c.v. administration of Aβ oligomers (Aβ + VEH) in absence or presence of FLX10, VTX5, or VTX10 on **(A)** iNOS and **(B)** Nox2 mRNAs expression measured by qRT-PCR. The abundance of each mRNA of interest was expressed relative to the abundance of GAPDH-mRNA, as an internal control. As a negative control, a reaction in absence of cDNA (no template control, NTC) was performed. qRT-PCR amplifications were performed at least in triplicate (mean of three to six determinations). Data are shown as mean ± S.E.M. *p < 0.05 vs. PBS + VEH, ^***^ *p* < 0.001 vs. PBS + VEH, ^#^ *p* < 0.05 vs. Aβ oligomers + VEH, ^###^ *p* < 0.001 vs. Aβ oligomers + VEH.

Oxidative stress and neuroinflammation play a significant role in the pathogenesis of depression (Bhattacharya and Drevets, 2017; Caruso et al., 2019a) and AD (Huang et al., 2016; Knezevic and Mizrahi, 2018). During the inflammation process, both inducible nitric oxide synthase (iNOS), responsible for nitric oxide production (Aktan, 2004; Metto et al., 2013), and NADPH oxidase 2 (Nox2), responsible for superoxide production (de Campos et al., 2015), are over-activated in immune cells including microglia (Siegel et al., 2019). When the above-mentioned enzymes are simultaneously activated, they synergistically promote neuronal cell death by generating peroxynitrite (Beckman and Crow, 1993). We therefore examined the effects of Aβ oligomers on the mRNAs levels of the pro-oxidant enzymes iNOS and Nox2 in the hippocampus (Figure 1), a brain area strongly affected in depression and AD (Villa et al., 2016; Setti et al., 2017). The i.c.v. injection of Aβ oligomers induced a statistically significant increase in the expression level of iNOS mRNA in the hippocampus compared with vehicle-treated controls (*p* < 0.05 vs. PBS + VEH; Figure 1A). Long-term i.p. treatment with FLX or VTX, administered at the same dose of 10 mg/kg, was able to abolish the over-expression of iNOS Aβ-induced (*p* < 0.05 vs. Aβ oligomers), whereas the lower dose of VTX (5 mg/kg) did not reach a statistically significant difference, even though a trend in iNOS mRNA enzyme expression decrease was observed. More robust effects were observed when measuring the variation of Nox2 mRNA expression levels under our experimental conditions. In fact, as shown in Figure 1B, the expression level of Nox2 mRNA was significantly increased in the hippocampus of Aβ-injected mice compared with vehicle-treated controls (*p* < 0.001 vs. PBS + VEH). Long-term i.p. treatment with FLX (10 mg/kg) or VTX, at both doses (5 or 10 mg/kg), was able to completely counteract the over-expression of this enzyme (*p* < 0.001 vs. Aβ oligomers for all of them). It is worth mentioning that treatment with FLX or VTX *per se* did not significantly modify the mRNA expression levels of iNOS and Nox2 enzymes (Supplementary Figure S1).

### Fluoxetine and Vortioxetine Decreased the Expression of iNOS and Nox2 Proteins

**FIGURE 2:**
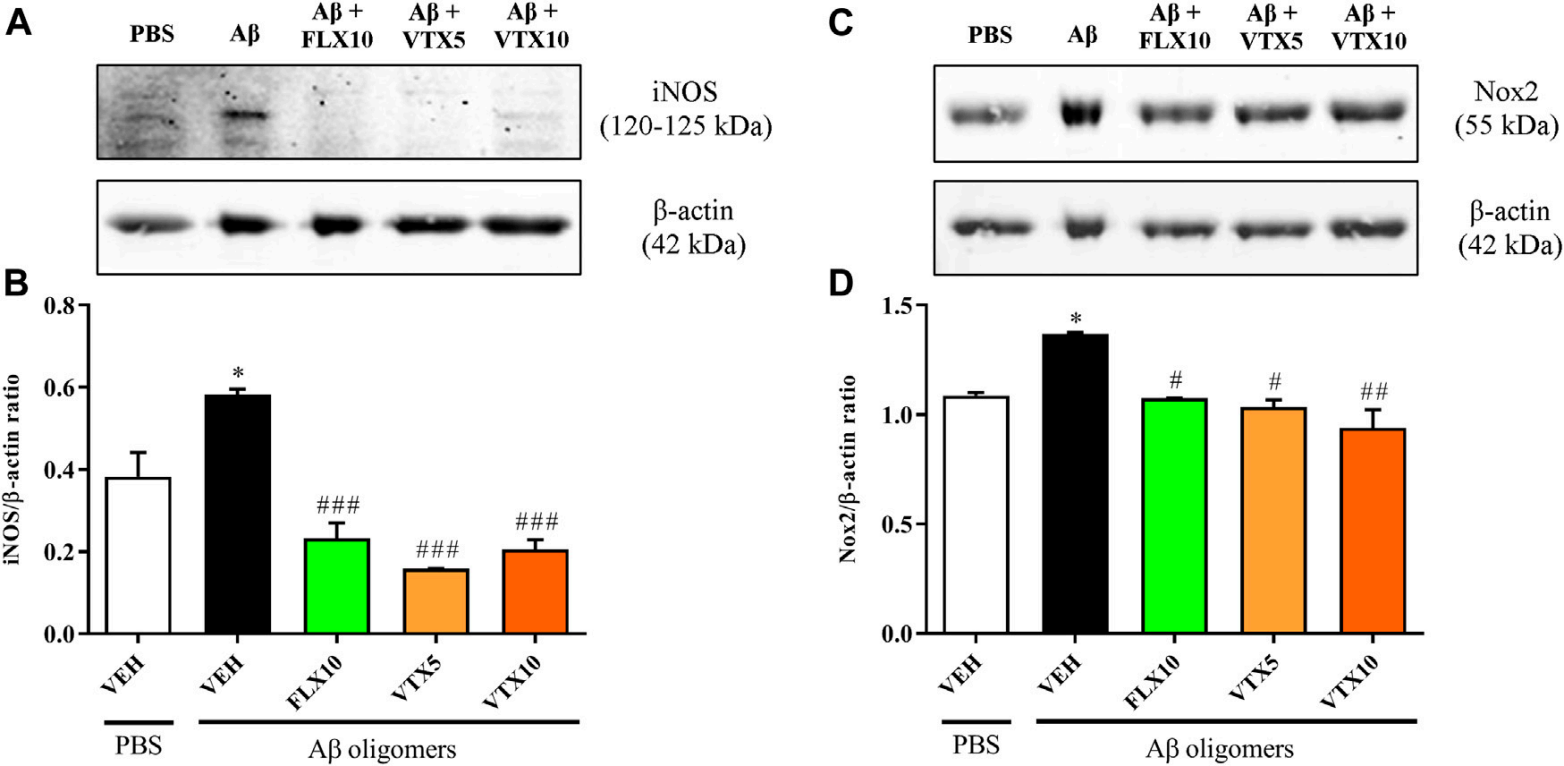
Fluoxetine and vortioxetine decrease the expression of iNOS and Nox2 proteins. Effects induced by i.c.v. administration of Aβ oligomers (Aβ + VEH) in absence or presence of FLX10, VTX5, or VTX10 on **(B)** iNOS and **(D)** Nox2 protein levels measured by WB. **(A, C)** show the representative immunoblots of iNOS (120–125 kDa) and Nox2 (55 kDa), respectively, in total protein extracts from hippocampal tissue. Histograms refer to the means ± S.E.M. of the densitometric values of iNOS or Nox2 bands normalized against β-actin (42 kDa). Each experiment was repeated three times. *p < 0.05 vs. PBS + VEH, ^#^ *p* < 0.05 vs. Aβ oligomers + VEH. ^##^ *p* < 0.01 vs. Aβ oligomers + VEH, ^###^ *p* < 0.001 vs. Aβ oligomers + VEH.

We then carried out WB experiments in order to corroborate with protein data the results obtained by using qRT-PCR in which the expression levels of iNOS and Nox2 mRNA were measured. WB analysis confirmed that the i.c.v. injection of Aβ oligomers is able to induce a significant increase of iNOS protein expression at hippocampal level (*p* < 0.05 vs. PBS + VEH) and, most importantly, that the treatment with FLX and VTX is able to completely abolish Aβ-induced iNOS expression (*p* < 0.001 vs. Aβ oligomers for both of them; Figure 2A). A very similar profile was observed when measuring the variation of Nox2 protein levels. Figure 2B shows that the expression level of Nox2 protein was significantly increased in the hippocampus of Aβ-injected mice compared with vehicle-treated controls (*p* < 0.05 vs. PBS + VEH). Treatment with FLX at the dose of 10 mg/kg or with the lower dose of VTX (5 mg/kg) abolished Aβ oligomers induction (*p* < 0.05 vs. Aβ oligomers), while a stronger decrease was observed in the case of the higher dose of VTX (10 mg/kg) (*p* < 0.01 vs. Aβ oligomers). As expected based on the qRT-PCR results, the treatment with FLX or VTX *per se* did not significantly modify the protein expression levels of iNOS and Nox2 enzymes (Supplementary Figure S2).

### Fluoxetine or Vortioxetine Rescued the Expression of Gpx1 at Gene but Not at Protein Level

**FIGURE 3:**
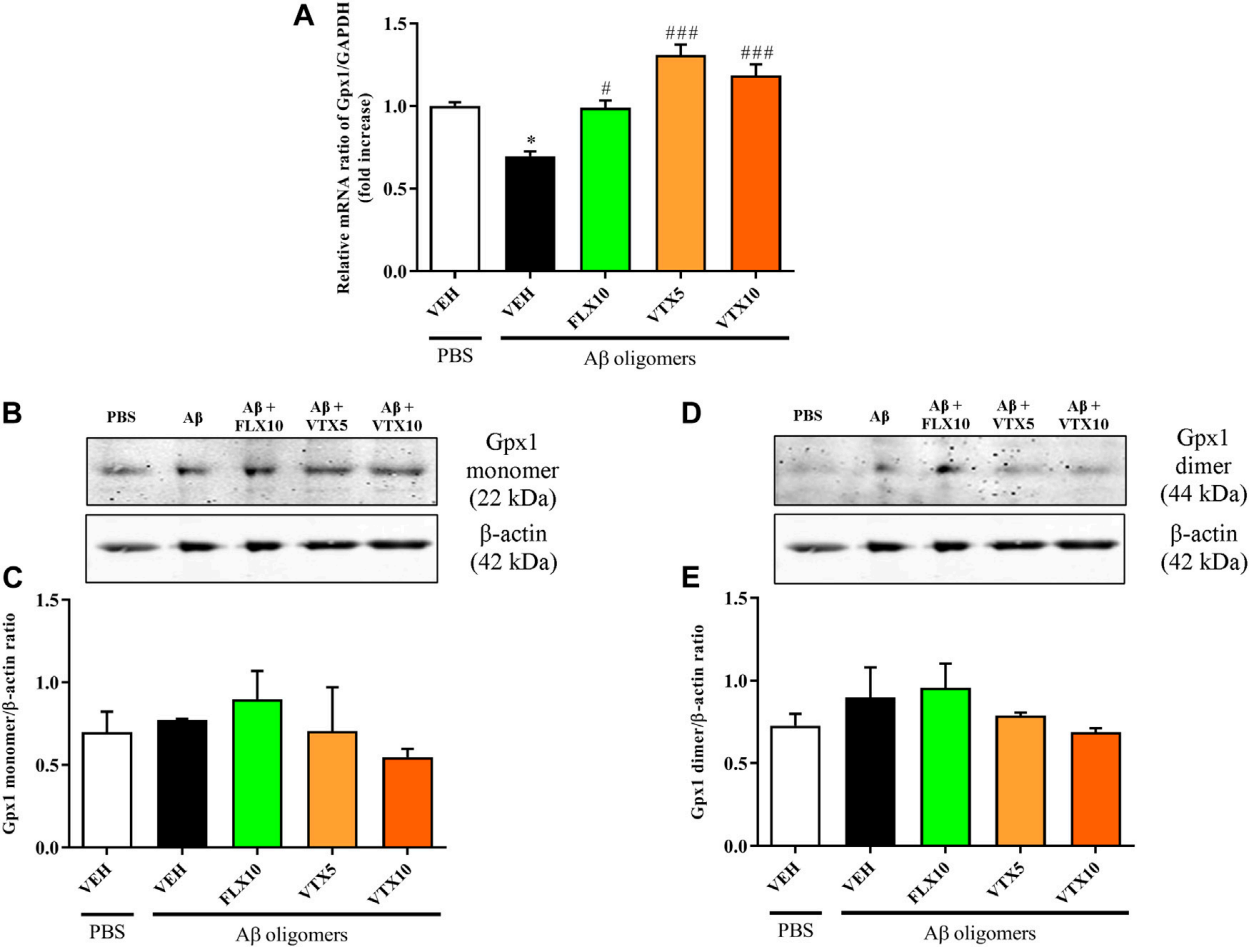
The treatment with fluoxetine or vortioxetine is needed to rescue the expression of Gpx1 at gene but not at protein level. Effects induced by i.c.v. administration of Aβ oligomers (Aβ + VEH) in absence or presence of FLX10, VTX5, or VTX10 on **(A)** Gpx1 mRNA expression measured by qRT-PCR; **(C)** monomer and **(E)** dimer of Gpx1 protein levels measured by WB. **(B, D)** show the representative immunoblots of Gpx1 monomer (22 kDa) and Gpx1 dimer (44 kDa) in total protein extracts from hippocampal tissue. In the case of gene expression measurements, the abundance of Gpx1 mRNA was expressed relative to the abundance of GAPDH-mRNA, as an internal control. As a negative control, a reaction in absence of cDNA (no template control, NTC) was performed. qRT-PCR amplifications were performed at least in triplicate (mean of three to five determinations). In the case of protein expression measurements, histograms refer to the means ± S.E.M. of the densitometric values of Gpx1 monomer or Gpx1 dimer bands normalized against β-actin (42 kDa). Each experiment was repeated three times. *p < 0.05 vs. PBS + VEH, ^#^ *p* < 0.05 vs. Aβ oligomers + VEH, ^###^ *p* < 0.001 vs. Aβ oligomers + VEH.

As previously mentioned, oxidative stress, a key factor in the progression of AD (Zhao and Zhao, 2013; Bajpai et al., 2014), reflects the imbalance between the production and quenching of reactive species in the biological system. This imbalance could also depend on the reduced activity of antioxidant enzymes such as glutathione peroxidase 1 (Gpx1) (Marcus et al., 1998; Katrenčíková et al., 2021). The lack of Gpx1 has been related to the exacerbation of Aβ-mediated neurotoxicity in cortical neurons (Crack et al., 2006). With this in mind, we investigated the effects of Aβ oligomers, in absence or presence of FLX10, VTX5, or VTX10, on the mRNA levels of Gpx1 in the hippocampus. The results depicted in Figure 3A show that the expression level of Gpx1 mRNA was significantly decreased in the hippocampus of Aβ-injected mice compared with vehicle-treated controls (*p* < 0.05 vs. PBS + VEH), while the treatment with FLX was able to completely restore Gpx1 mRNA levels (*p* < 0.05 vs. Aβ oligomers). A more significant effect was observed in the case of both doses of VTX (*p* < 0.001 vs. Aβ oligomers). Since it has been shown that oxidative stress can induce multimerization of Gpx1 enzyme by forming complexes via oxidative linkage between subunits (Park et al., 2004; Sultan et al., 2018), we performed WB analysis by measuring both monomeric and dimeric forms. However, the results obtained by WB showed that either the monomeric nor the dimeric form of Gpx1 protein in the hippocampus were significantly affected by Aβ treatment as compared to vehicle-treated controls (Figures 3B,C). The presence of antidepressants during Aβ treatment did not modulate the expression levels of either enzyme isoforms. The treatment with FLX or VTX *per se*, in the absence of Aβ treatment, did not significantly modify the mRNA (Supplementary Figure S1) and protein (Supplementary Figure S2) levels of Gpx1 enzyme.

### Fluoxetine and Vortioxetine Exert Neuroprotection Against the Toxicity Induced by Aβ Oligomers

**FIGURE 4:**
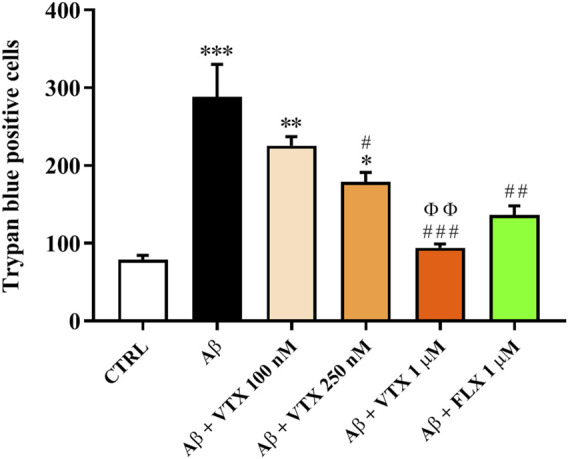
Fluoxetine and vortioxetine exert neuroprotection against the toxicity induced by Aβ oligomers. Primary mixed neuronal cultures were treated with Aβ oligomers (2 µM) for 48 h both in absence or presence of FLX (1 µM) or increasing concentrations of VTX (100 nM, 250 nM, and 1 µM) (pre-treatment of 1 h). The toxicity of Aβ oligomers in mixed neuronal cultures was assessed by cell counting after trypan blue staining. Cell counts was performed in three random microscopic fields/well. Data are the mean of three to four determinations. S.D. are represented by vertical bars. *p < 0.05 vs. CTRL, **p < 0.01 vs. CTRL, ***p < 0.001 vs. CTRL, ^#^ *p* < 0.05 vs. Aβ, ^##^ *p* < 0.01 vs. Aβ, ^###^ *p* < 0.001 vs. Aβ, ^ՓՓ^ *p* < 0.01 vs. VTX 100 nM.

The neuroprotective activity of FLX in mixed cultures of cortical cells treated with Aβ oligomers, representing an established experimental model of Aβ-induced neurodegeneration (Caruso et al., 2019c), has been already reported (Caraci et al., 2016). However, it is presently unknown whether a treatment with VTX can prevent the neuronal cell death due to Aβ treatment. We then investigated the neuroprotective activity of increasing concentrations of VTX in mixed cultures of cortical cells treated with Aβ oligomers (2 µM) for 48 h. In this set of experiments, FLX at the concentration of 1 µM was used as a gold standard. Since Aβ is known to promote glutamate release and toxicity (Caraci et al., 2011), the experiments were performed in the presence of a cocktail of ionotropic glutamate receptor antagonists [MK-801 (10 µM) and DNQX (30 µM)] to exclude the contribution of endogenous excitotoxicity to the overall process of neuronal death. The treatment of mixed cultures of cortical cells with Aβ oligomers for 48 h led to a significant increase (about 300%) in the number of trypan blue positive cells (dead neurons) compared to untreated (CTRL) cells (*p* < 0.001) (Figure 4). VTX, starting at a concentration of 250 nM, significantly prevented Aβ toxicity in mixed neuronal cultures (*p* < 0.05 vs. Aβ), though not completely. The maximal neuroprotective effect was observed in the case of VTX 1 µM (*p* < 0.001 vs. Aβ), with a number of dead cells comparable to that observed for untreated cells or for FLX-treated cells (positive control).

## Discussion

Reactive species such as reactive oxygen species (ROS) and reactive nitrogen species (RNS) play a crucial role in numerous human pathophysiological processes. These species, when produced at physiological concentration, are able to regulate growth, apoptosis, and complex functions such as blood pressure, immune and cognitive functions. When overproduced, they can contribute to a well-known and deleterious phenomenon called oxidative stress (Estévez and Jordán, 2002). During acute oxidative stress, the components of the antioxidant system are able to counteract the increased levels of pro-oxidants, such as ROS and RNS, resetting them to the physiological levels. Whenever ROS and RNS levels overcome the antioxidant defense, chronic oxidative stress takes place, as it has been observed in neuropsychiatric disorders such as depression and AD (Caruso et al., 2019d). Both ROS and RNS are involved in the pathogenesis of depression by influencing neuronal processes such as neurogenesis and neuroplasticity, also inducing neuroinflammation and neurodegeneration (Cheignon et al., 2018; Solleiro-Villavicencio and Rivas-Arancibia, 2018). When considering AD, the over-production of ROS and RNS, and then oxidative stress, has been related to increased Aβ production and/or aggregation, which in turn exacerbates neuronal oxidative damage, contributing to neuronal death in AD brain (Cheignon et al., 2018).

In the present work we employed a non-Tg model of AD, obtained by i.c.v. injection of Aβ oligomers, allowing to investigate the molecular mechanisms through which the oligomeric form of Aβ causes cognitive dysfunction, and finally to test novel pharmacological approaches (Balducci and Forloni, 2014). As previously demonstrated, this non-Tg AD model is characterized by cerebral concentrations of soluble Aβ oligomers comparable to those observed in the AD brain, sufficient to induce a memory deficit that persists for 2–3 weeks (Leggio et al., 2016). By adopting this model, we have recently demonstrated that both FLX and VTX possess the ability to reverse the depressive-like phenotype and memory deficits induced by the i.c.v. injection of Aβ oligomers in mice, also rescuing the levels of the synaptic proteins synaptophysin and PSD-95 as well as of TGF-β1, the deficit of which has been shown to contribute to inflammation and cognitive decline both in depression and AD (Caraci et al., 2018b; Torrisi et al., 2019). In order to correlate the above-mentioned preclinical efficacy of these two second-generation antidepressants with an antioxidant activity of these drugs, we examined the mRNA levels of iNOS and Nox2, two pro-oxidant enzymes synergistically able to promote neuronal death through the production of ROS and RNS (Beckman and Crow, 1993), in the hippocampus of Aβ-injected mice, a brain area strongly affected in both depression and AD (Villa et al., 2016; Setti et al., 2017). Our results show that the i.c.v. injection of Aβ oligomers in mice induced a significant increase in iNOS and Nox2 enzymes in the hippocampus, both at mRNA (Figure 1) and protein (Figure 2) level, compared with vehicle-treated controls. These findings are in agreement with an *in vivo* study carried out by Medeiros et al. (2007) in which the i.c.v. injection of Aβ1–40 induced iNOS protein expression in hippocampus and prefrontal cortex of mice that was paralleled by marked deficits of learning and memory, emphasizing the deleterious effects of aberrant expression of NOS isoforms in AD brain (Lüth et al., 2002). Our findings are also in line with other studies showing that AD is characterized by inflammatory processes in which the production of nitric oxide from iNOS and/or superoxide from Nox2 is strongly increased (Murphy, 2000; Brown and Bal-Price, 2003; Zekry et al., 2003). In the present study we showed for the first time that a long-term i.p. treatment with FLX or VTX was able to abolish the over-expression of iNOS and Nox2 induced by Aβ oligomers (Figures 1, 2). These results are also consistent with previous findings, showing the ability of antidepressant drugs to exert immune-regulatory effects by reducing the levels of pro-inflammatory cytokines, also decreasing the production of reactive species and/or enhancing key elements of the antioxidant machinery. Along this line, FLX has been shown to revert brain oxidative damage by reducing lipid peroxidation and increasing the activity of the antioxidant enzymes (i.e., CAT and SOD) in the hippocampus of an animal model of depression (Moretti et al., 2012). In a study carried out by Talmon et al. (2018) the multimodal antidepressant VTX was able to reduced the oxidative burst induced by PMA in monocytes and in macrophages (M1 and M2), also leading to a shift of macrophage polarization from the pro-inflammatory (M1) to the anti-inflammatory (M2) phenotype. The above reduction of oxidative stress was also paralleled by a decrease of nuclear factor kappa-light-chain-enhancer of activated B cells (NF-kB) translocation and TNF-α release. The present data on the antioxidant activity of FLX and VTX *in vivo* reinforce the above-described previous findings obtained in translational models of inflammation. Our findings on the antioxidant activity of FLX and VTX might be therapeutically relevant in the context of depression and AD, when considering that depression acts as a risk factor for AD and, most importantly, that oxidative stress processes play a key role in the pathophysiology of cognitive deficits in depression (Scapagnini et al., 2012).

About 30% of depressed patients show a poor response to conventional antidepressants associated with a significant cognitive impairment and a poor quality of life, a clinical phenotype classified as treatment-resistant depression (TRD) (Caraci et al., 2018a). It has been recently shown how TRD is characterized by increased oxidative stress coupled to inflammation (Sowa-Kućma et al., 2018). Furthermore, plasma levels of Coenzyme Q10 (CoQ10), a strong antioxidant with anti-inflammatory activity, are lower in TRD patients compared to responders depressed patients (Maes et al., 2009) and CoQ10 (200 mg/die) has been proposed as an adjuvating agent for the treatment of depression (Mehrpooya et al., 2018). According to this scenario second-generation antidepressant drugs, endowed with antioxidant activity, may display an increased clinical efficacy in TRD patients; studies in animal models are useful to test this hypothesis. As previously mentioned, antioxidants play a significant role in maintaining redox homeostasis. It is also worth mentioning that an increase in Nox2 protein expression and the related oxidative stress have also been observed in sleep deprivation known to induce memory impairments, serotoninergic system dysfunction, and depression in mice (Wang et al., 2020). In our experimental model of amyloid-related depression the presence of depressive-like behavior and memory deficit was paralleled by an increase in Nox2 protein levels in Aβ-injected mice that was rescued by the long-term treatment with FLX at 10 mg/kg and more significantly by VTX, being effective at the lowest dose of 5 mg/kg, suggesting that the antidepressant activity of these drugs is not simply related to the inhibition of serotonin transporter (SERT), but it is also includes antioxidants effects.

In our study we also measured the mRNA and protein levels of Gpx1, an antioxidant enzyme known to play a protective role against Aβ-toxicity and the related ROS accumulation at intracellular level (Barkats et al., 2000), and the deficit of which has been related to increased Aβ-mediated neurotoxicity (Crack et al., 2006). Our data show that the treatment with FLX or VTX is needed to rescue the expression of Gpx1 at mRNA, but not at protein level. We cannot exclude a post-trascriptional regulation of Gpx1 gene expression. In our case, the discrepancy observed when comparing mRNA and protein levels within the same time frame might be due to a compensatory response of the antioxidant system with an additional role played by FLX or VTX, which remains to be elucidated.

We know from our previous work in the non-Tg model of AD that FLX and VTX not only counteract oxidative stress, but also rescue the hippocampal TGF-β1 levels. This neurobiological link could be of utmost importance since it has already been demonstrated that other multimodal drugs that counteract oxidative stress can also rescue TGF-β1 levels (Caruso et al., 2019b; Fresta et al., 2020b). In two very recent research studies it has also demonstrated the ability of TGF-β1 to protect retinal ganglion cells against oxidative stress through the modulation of the HO-1/Nrf2 pathway (Chen et al., 2020) and chondrocytes *via* FOXO1-autophagy axis (Kurakazu et al., 2021), strongly suggesting a key role of TGF-β1 in counteracting oxidative stress induced by Aβ.

In the AD brain, oxidative stress promotes the generation of ROS that contribute to neurodegeneration (Shimohama et al., 2000; Abramov and Duchen, 2005). During the last decade, based on their ability to exert neuroprotection, the use of antidepressants to reduce the risk to develop AD has been proposed (Kessing et al., 2009; Kessing, 2012; Yuste et al., 2015; Bartels et al., 2018). By using a well-established *in vitro* model of Aβ-induced neurodegeneration consisting of mixed cultures of cortical cells challenged with Aβ oligomers, we were able to compare, for the first time, the well-known neuroprotective activity of FLX (Caraci et al., 2016) with the protective effects of VTX. Interestingly we found that VTX pre-treatment started to exert significant neuroprotective effects at nanomolar concentrations (250 nM) with a maximal effect at 1 μM, similar to that observed for FLX pre-treatment and untreated cells (Figure 4). The neuroprotective effects exerted *in vitro*, as well as the antidepressant effects exerted *in vivo* by FLX and VTX could be related to the anti-amyloidogenic and anti-aggregant activity of these drugs. In particular, SSRIs including FLX have shown the potential to prevent Aβ aggregation by direct binding and could be beneficial to AD patients (Tin et al., 2019). FLX also possesses a recognized ability to revert soluble Aβ-induced depressive phenotype with a specific “Aβ-lowering” effect (Schiavone et al., 2017). Furthermore, it cannot be excluded that both FLX and VTX could exert their therapeutic potential by enhancing the release of TGF-β1 from microglial cells, as observed in our *in vivo* experiments, then rescuing the antioxidant system through the activation of TGF-β1 signaling. The results observed in our experimental models with therapeutic concentrations of VTX also stimulate further studies both in rodents and MCI patients with amyloid-related depression.

All together the data presented in this study, obtained by using a non-Tg model of AD, demonstrated that oxidative stress, taking place as a consequence of pro-oxidant enzymes (i.e., iNOS and Nox2) activation, along with the previously showed deficit of TGF-β1, represents one of the neurobiological links between depression and AD. We also showed for the first time how a long-term administration of the antidepressants fluoxetine and vortioxetine, able to rescue the TGF-β1 pathway, can also contribute to prevent amyloid-induced depression and cognitive decline by counteracting Aβ-induced oxidative stress.

## Data Availability

All datasets generated for this study are included in the article and the Supplementary Material. Further inquiries can be directed to the corresponding author.
