# Supplementary material for: Antioxidant Activity of Fluoxetine and Vortioxetine in a Non-Transgenic Animal Model of Alzheimer’s Disease
Source: Front Pharmacol. 2021 Dec 24;12:809541. doi: 10.3389/fphar.2021.809541 (PMC8740153; doi:10.3389/fphar.2021.809541)
Supplement: Supplementary file 1 [file DataSheet1.docx]

Supplementary Material

**Supplementary Figure 1.** **Fluoxetine and vortioxetine do not influence the basal expression levels of iNOS, Nox2, and Gpx1 mRNAs**. Effects induced by drugs administration in absence of Aβ oligomers treatment on A) iNOS, B) Nox2, and C) Gpx1 mRNAs expression measured by qRT-PCR. The abundance of each mRNA of interest was expressed relative to the abundance of GAPDH-mRNA, as an internal control. As a negative control, a reaction in absence of cDNA (no template control, NTC) was performed. Data are shown as mean ± S.E.M. of three to six independent experiments.

**Supplementary Figure 2.** **Fluoxetine and vortioxetine do not influence the basal expression levels of iNOS, Nox2, and Gpx1 proteins**. Effects induced by drugs administration in absence of Aβ oligomers treatment on A) iNOS, B) Nox2, C) Gpx1 monomer, and D) Gpx1 dimer protein **levels measured by WB.** Histograms refer to the means ± S.E.M. of three independent experiments. The densitometric values of iNOS or Nox2 or Gpx1 monomer or Gpx1 dimer bands were normalized against β-actin.
